# Scabies-infested pregnant women: A critical therapeutic challenge

**DOI:** 10.1371/journal.pntd.0008929

**Published:** 2021-01-07

**Authors:** Amandine Weill, Charlotte Bernigaud, Mourad Mokni, Sophie Gil, Elisabeth Elefant, Olivier Chosidow

**Affiliations:** 1 Service de Dermatologie, Hôpital Henri Mondor, AP-HP, Université Paris-Est, Créteil, France; 2 Groupe de recherche Dynamyc, EA7380, Faculté de Santé de Créteil, École nationale vétérinaire d’Alfort, USC ANSES, Université Paris-Est Créteil, Créteil, France; 3 Fondation PremUp, Paris, France; 4 Service de Dermatologie, Hôpital La Rabta, Faculté de Médecine, Université al Manar 2, Laboratoire de Recherche Infection et Santé Publique LR18SP01, Tunis, Tunisie; 5 Université de Paris, INSERM, UMR-S 1139, 3PHM, Paris, France; 6 Centre de Référence sur les Agents Tératogènes (CRAT), Hôpital Armand Trousseau, AP-HP, Sorbonne Université, Paris, France; Tulane University, UNITED STATES

## Pregnancy: A neglected condition in the management of scabies

Scabies is a global public health burdensome issue with an estimated worldwide point prevalence of up to 200 million people in 2015 according to the Global Burden of Disease Study [1,2]. Pregnant women are at increased risk of certain infectious diseases, such as influenza, malaria, hepatitis E, measles, smallpox, and herpes simplex virus infection, potentially owing to a weakened adaptive immune response [3,4]. Yet, the susceptibility to scabies has not been specifically investigated in pregnant women, and scabies accounts for 2% to 6% of all pregnancy skin diseases according to observational studies [5,6]. Besides, the prevalence of this neglected tropical disease (NTD) is particularly high in low- and middle-income countries [2], where women of reproductive age face unmet needs for contraceptives: up to 20% of women become pregnant before the age of 18 years (https://www.unfpa.org). Along with psychosocial and economic impacts, the global burden of scabies caused by major sleep disturbance and skin damage related to scratching, streptococcal and staphylococcal superinfections (with increased risk of fatal invasive sepsis in impoverished countries) and post-streptococcal complications (glomerulonephritis, acute rheumatic fever, or rheumatic heart disease) [1] may not differ during pregnancy.

To date, scarce data specifically related to treating scabies infestation in pregnant women are available. The therapeutic safety and efficacy of scabicides were recently reviewed in 2 Cochrane systematic reviews that included 22 and 15 randomized controlled trials (RCTs) [7,8], the first included 17 RCTs involving women of reproductive age [7]. Yet, the exclusion of pregnant women was systematic in 11 of the RCTs and not stated in 3. In the systematic review by Rosumeck and colleagues [8], only 1 RCT of patients aged 5 to 15 years did not exclude pregnant women, and in another, exclusion criteria were not stated. The low proportion of pregnant women leads to a limitation of the external validity to this population. Public sources of information and advice for the use of scabicide drugs during pregnancy are available on the website of the United Kingdom Teratology Service Information (https://www.medicinesinpregnancy.org), but the site does not discuss the question of the safety of oral ivermectin for scabies-infested pregnant women.

## Treatment resource shortage in scabies-infested pregnant women: Current therapeutic guidelines and limitations

Currently, only topically applied scabicide drugs are available for treating scabies infestation in pregnant women in most countries. According to the systematic reviews, 5% permethrin (ranked B in the United States Food and Drug Administration pregnancy category https://www.fda.gov) is considered the reference topical treatment and the treatment of choice during pregnancy [8]. Animal studies did not evidence fetal harm or mutagenicity. With prospective comparison to a control group, 113 pregnant women exposed to permethrin (31 during the first trimester) did not differ in spontaneous abortions, malformations, birth weight, or gestational age at delivery [9]. Second-line (benzyl benzoate 25% lotion and precipitated sulfur) and third-line (crotamiton and 0.5% malathion) treatments are less effective than permethrin, and safety data of these drugs are limited [7,10]. Animal studies have not been conducted for precipitated sulfur. Human in vivo safety data for benzyl benzoate mainly relies on a retrospective study of 640 pregnant women exposed to benzyl benzoate (*n* = 444) or permethrin (*n* = 196), with only 10.9% (*n* = 66) exposed during the first trimester [11]. No studies have investigated crotamiton (category C), and a possible association with shortened gestational duration was evidenced with malathion (category B) [10].

Oral ivermectin, part of the 21st List of Essential Medicines according to the World Health Organization and widely used, is currently the only systemic treatment available to cure scabies as effectively as permethrin [8]. Nevertheless, it is ranked C by the United States Food and Drug Administration and remains contraindicated for pregnant women in most countries. These basic precautions mainly rely on experimental studies in mice showing adverse pregnancy outcomes (oral clefts and clubbed forepaws) at doses 10 to 100 times higher than current ivermectin human doses (150 to 200 μg/kg) (https://www.merck.ca).

Consequently, scabies treatment options in pregnancy remain limited and nonideal and have many limitations. The binding therapeutic regimen of topical scabicides—applied from head to toe and repeated twice a week apart—is impractical and not sustainable, especially in tropical outbreak areas where cream applications are associated with discomfort and are improperly used in case of insufficient water facilities [12]. Furthermore, when applied on damaged skin, topical agents carry the risk of serious side effects such as systemic exposure, which indeed led to the withdrawal of topical lindane from European and United States markets in 1998, owing to maternal neurotoxicity and aplastic anemia. Moreover, cutaneous side effects (e.g., stinging and burning) of topical agents are associated with poor compliance, which hampers the control of such a global disease.

**Fig 1:**
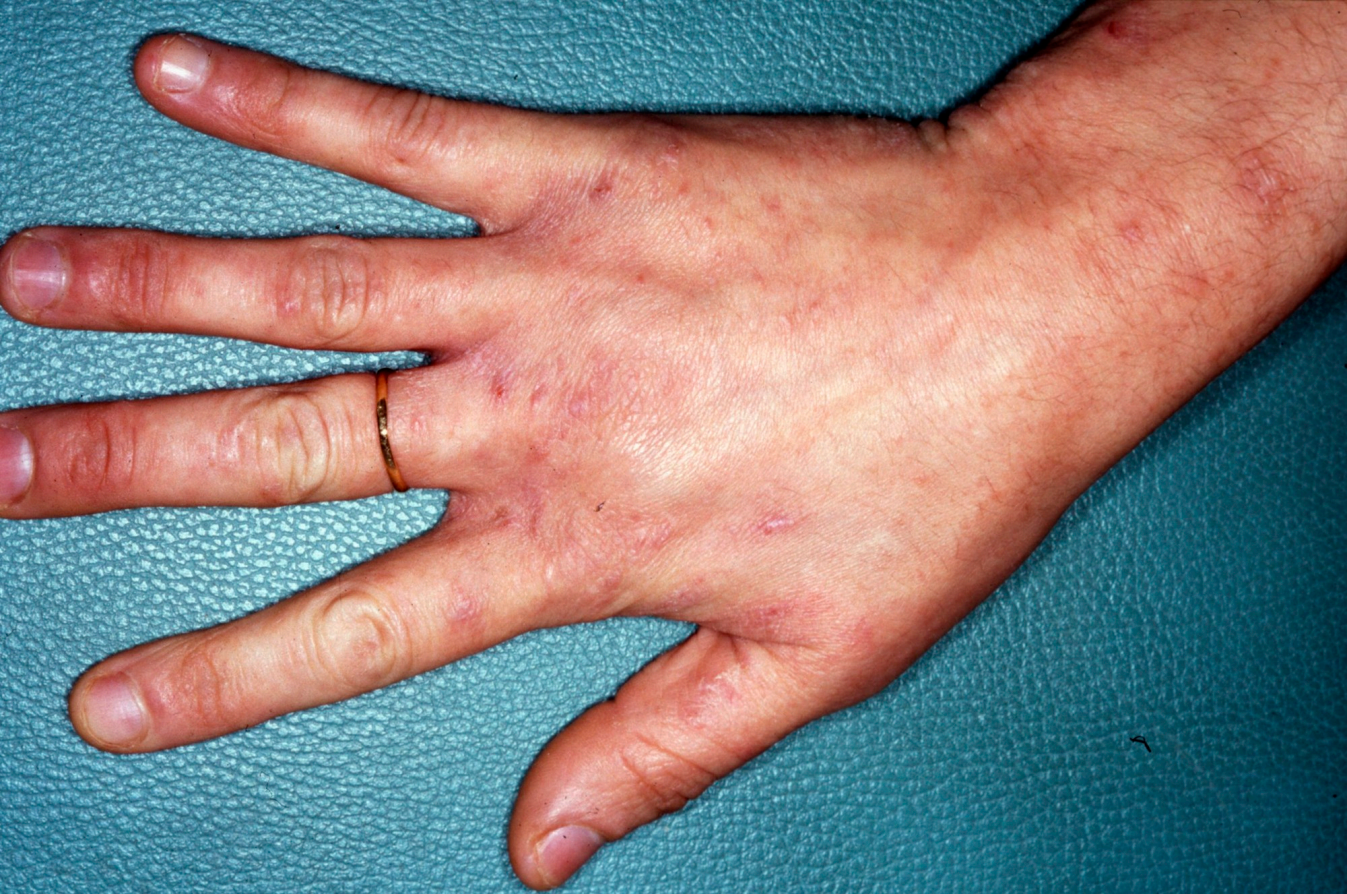
Hand of a scabies-infested pregnant woman with diffuse damaged skin. A 38-year-old Tunisian woman at 16 weeks of pregnancy presenting scabies as did her husband and their 2 children. Scabies was present for 4 months, and the skin was largely damaged with widespread eczematous on the limbs, trunk, breast, and nipples. Therapeutic management was challenging because topical scabicides were inconceivable, and oral ivermectin was unavailable in Tunisia. Oral ivermectin, 200 μg/kg body weight, repeated 1 week apart and brought back from the European market by the dermatologist, finally allowed for effective and safe treatment without any adverse pregnancy outcome. *Collection of Prof*. *Mourad Mokni (MD*, *PhD)*, *Department of Dermatology*, *La Rabta Hospital*, *Tunis*, *Tunisia*.

Thus, how should we treat scabies infestation in pregnant women with diffuse, eczematous, superinfected, and/or damaged skin, with any proper application of topicals being unrealistic (Fig 1)?

## Safety of ivermectin during pregnancy: From populational data to decision-making

From our French experience, we believe that access to ivermectin should be safely widened to scabies-infested pregnant women to solve such challenging and not unusual situation.

In line with the expertise of the “Centre de Référence sur les Agents Tératogènes” Reference Center on Teratogenic Agents (CRAT, https://www.lecrat.fr)—the first and largest organization devoted to the safety of drugs during pregnancy worldwide—France is the only country where ivermectin use is considered for treating scabies during pregnancy, as second-line treatment after permethrin or as first-line treatment combined with permethrin if needed. These recommendations are based on retrospective data from inadvertent pregnancy exposure to ivermectin, as part of onchocerciasis mass drug administration programs in sub-Saharan Africa and ivermectin pharmacological properties. As recently reviewed in a systematic review by Nicolas and colleagues [13], birth outcomes are not excessively abnormal in ivermectin-treated compared with that in unexposed women. Although few studies addressed the safety of ivermectin during pregnancy, they still involved 899 pregnancy birth outcomes and almost 100 pregnant women exposed during their first trimester (allowing the assessment of congenital abnormalities). These studies gathered a rather significant absolute number of cases for which we believe that the certainty of evidence might not be considered “very low.” Furthermore, the selection biases discussed in these observational studies are not obvious or concerning, and there is no specific recall bias as evidenced by the lack of increased congenital abnormalities rate between groups. Yet, these studies were not intended to assess the fetal safety of ivermectin exposure during pregnancy, and some imprecisions on the term “ivermectin intake” may exist. Further studies could be useful. Nevertheless, RCTs of first-trimester pregnant women are not feasible for ethical reasons. Further open data repositories have been proposed but raise doubt as to their ability to conclude [13]. Yet, regarding the available data and the likely benefit and cost-effectiveness of ivermectin in scabies and other parasitic NTDs (onchocerciasis and filariasis), we suggest aligning with the CRAT recommendations and support the second-line use of ivermectin in scabies-infested pregnant women. Yet, a postponement until the end of organogenesis (e.g., 10 weeks of amenorrhea) could be an interesting precaution whenever possible.

## What could be the future of oral scabicide drugs?

### International guidelines should include cases of scabies-infested pregnant women

As mentioned above, in the absence of devoted controlled studies, treatment recommendations for scabies during pregnancy mainly rely on animal studies, published clinical experience, and expert opinions [10]. To our best knowledge, neither French nor global pharmacovigilance signals referring to oral ivermectin during pregnancy have been published since its launch on the market in 2001 (https://ansm.sante.fr, https://www.who-umc.org). Efforts should be implemented to promote the collection of data to evaluate the true burden of scabies during pregnancy and further assess its optimal management depending on clinical presentation and area of the world. Such steps could be encouraged and facilitated with the support of other expert groups such as the International Alliance for the Control of Scabies (https://www.controlscabies.org) and the World Health Organization.

### Widening the access to oral ivermectin by scabies-infested pregnant women?

To support reassuring in vivo data, understanding the pharmacological properties of ivermectin—substrate of P-glycoprotein efflux transporters encoded by the *ABCB1* gene—could also be useful to expand our knowledge. In mammals, P-glycoproteins keep ivermectin from crossing the blood–brain barrier, blocking ivermectin-induced neurotoxicity [14]. Neurological disorders after ivermectin intake are uncommon in humans and could be linked to rare *ABCB1*-nonsense mutations leading to nonfunctional P-glycoproteins if mutated on both alleles [15]. P-glycoproteins are also expressed on the maternal side of the placenta and were found to prevent potential harmful xenobiotics to reach to the fetus in ex vivo human study [16]. Yet, this effect has not been evidenced for ivermectin in human placenta. Experimental studies using the human ex vivo model “placental perfusion” could provide further reassuring data on the transplacental transfer (TPT) of ivermectin in humans [17]. Indeed, this experimental method is the only model that simultaneously restores the maternal and fetal circulation. It allows for assessing the drug TPT, its mechanisms of transfer, and the function of efflux transporters (such as P-glycoproteins) under controlled conditions [17]. This ex vivo model would be convenient for studying ivermectin TPT because it does not raise ethical problems, placentas being considered waste. Yet, maternal informed consent might be easily obtained, either before the delivery at any obstetrical visit or shortly after the collection of the placenta. Furthermore, the model has been successfully used to predict the TPT of different molecules (e.g., HIV protease inhibitors or taxanes used in breast cancers), with good correlation between in vivo data and perfusion model [18].

### What about moxidectin during pregnancy?

Moxidectin—another oral macrocyclic lactone with a longer half-life than ivermectin and recently licensed for the treatment of onchocerciasis—represents a promising oral cure for human scabies and is currently being investigated in a phase II study (NCT03905265) [12,19]. Determining its safety during pregnancy should be a priority if it is ever used in mass drug administration programs and as a convenient resource for scabies-infested pregnant women in the future.

## Is lactation possible for scabies-infested pregnant women and is breastfeeding safe while using scabicide drugs?

In adults, scabies lesions are often clustered on the breasts and nipples. This situation raises the question of both the risk of mother-to-child transmission of scabies and scabicide drug absorption through breastfeeding. Scabies-infested lactating women should express their milk as long as they are not receiving an effective treatment, which allows for maintaining maternal breast milk feeding and limiting the risk of scabies transmission to the infant. For pregnant women, few data are available concerning the safety of scabicide drugs use during lactation. Excretion of permethrin and benzyl benzoate in breast milk after topical application has not yet been assessed, but permethrin residues in breast milk were evidenced after extensive exposure from agricultural use or malaria control in 1 study [20]. Regarding the very low absorption rate of permethrin after topical administration (2%), its rapid metabolism to inactive metabolites, and its safe application on infant skin, 5% permethrin cream is considered safe for nursing mothers and should be preferred over benzyl benzoate [21]. Yet, breastfeeding might be withheld during the 8 hours of permethrin topical application. Excretion of ivermectin in human breast milk was evaluated in 1 study of 4 lactating non-breastfeeding women after ivermectin intake at 150 μg/kg [22]. The authors indicated that a 1-month-old child would receive a dose of approximately 2.75 μg/kg (i.e., 1% of the maternal dose) on the day of the drug intake [22]. Also, from their 3 years of experience using ivermectin in onchocerciasis endemic areas, with lactating mothers constituting 5% to 10% of the population, no serious side effects were observed [22]. Both the American Academic of Pediatrics (https://www.aap.org) and the CRAT consider ivermectin compatible with breastfeeding.

## Conclusion

In scabies-infested pregnant women, topical permethrin remains the first-line treatment. However, in some situations (e.g., scabies with eczematous or superinfected skin), oral ivermectin represents the only alternative, which is supported by CRAT guidance. Innovating models such as the human ex vivo model “placental perfusion” and further recommendations from expert groups in the field of scabies and drug-induced teratology would help promote a wider access to oral scabicides by pregnant women.
